# The Role of Visible and Infrared Spectroscopy Combined with Chemometrics to Measure Phenolic Compounds in Grape and Wine Samples

**DOI:** 10.3390/molecules20010726

**Published:** 2015-01-07

**Authors:** Cozzolino Daniel

**Affiliations:** School of Agriculture, Food and Wine, Faculty of Sciences, The University of Adelaide, Waite Campus, PMB 1 Glen Osmond, Adelaide, SA 5064, Australia; E-Mail: d.cozzolino@adelaide.edu.au; Tel.: +61-8-8313-6738; Fax: +61-8-8313-7129

**Keywords:** phenolics, grapes, wine, near infrared, mid infrared, visible, spectroscopy

## Abstract

The content of phenolic compounds determines the state of phenolic ripening of red grapes, which is a key criterion in setting the harvest date to produce quality red wines. Wine phenolics are also important quality components that contribute to the color, taste, and mouth feel of wines. Spectroscopic techniques (e.g., near and mid infrared) offer the potential to simplify and reduce the analytical time for a range of grape and wine analytes. It is this characteristic, together with the ability to simultaneously measure several analytes in the same sample at the same time, which makes these techniques very attractive for use in both industry and research. The objective of this mini review is to present examples and to discuss different applications of visible (VIS), near infrared (NIR) and mid infrared (MIR) to assess and measure phenolic compounds in grape and wines.

## 1. Introduction

Phenolic compounds, abundant in plants, are of considerable interest and have received more and more attention in recent years due to their bioactive functions [1,2,3,4]. Polyphenols are amongst the most desirable phytochemicals due to their antioxidant activity. These components are known as secondary plant metabolites, having antimicrobial, antiviral and anti-inflammatory properties along with their high antioxidant capacity [1,2,3,4]. Efforts have been made to develop highly sensitive and selective analytical methods for the determination and characterisation of polyphenol compounds where extensive research has been conducted on the different extraction and separation methods or techniques, as well as improving the chromatographic and spectral techniques used (e.g., NMR, infrared, UV, visible spectroscopy) [1,2,3,4].

Wine phenolics are important quality components that contribute to the color, taste, and mouth feel of wines [2,3,4,5]. Although phenolic compounds found in wine can also originate from microbial and oak sources, the majority of the phenolic constituents found in wine are grape-derived [1,2,3,4,5]. Grape growers and winemakers inherently understand the importance of grape and wine phenolics to overall wine quality, yet increasingly, few advances in the understanding of grape and wine phenolic chemistry have been made [2,3,4,5]. Wine is mainly composed of water, alcohol and other minor chemical components such as proteins, sugars, phenolic and volatile compounds that are present at low concentration (mg/100 g) [6,7,8,9,10,11,12]. The content of phenolic compounds determines the state of phenolic ripening of red grapes and is a key criterion in setting the harvest date to produce quality red wines. Grape and wine phenolics are structurally diverse, from simple molecules to oligomers and polymers usually designated as tannins [2,3,4,5]. They have an important impact on the organoleptic properties of wines; that is why their analysis and quantification are of primordial importance. The extraction of phenolics from grapes and from wines is the first step involved in analysis followed by several analytical methods that have been developed for the determination of total content of phenolic, while chromatographic and spectrophotometric analyses are continuously improved in order to achieve adequate separation of phenolic molecules, their subsequent identification and quantification [2,3,4,5]. The phenolic composition of grapes at the harvest time is a key factor determining their quality, and thus the quality of the finished wine [2,3,4,5]. The chemical methods used for the determination of seed and skin phenol content and extractability are generally slow because they require a preliminary extraction. Therefore, an evaluation of these parameters could be highly interesting for the oenological sector [6,7,8,9,10,11,12].

Today, public demand for high levels of quality and safety in grape and wine production requires high standards in quality assurance and process control methods, and this demand in turn requires appropriate tools for analysis during and after production. Desirable features of such tools should include speed, ease-of-use, minimal or no sample preparation, and the avoidance of sample destruction. These features are the main characteristics of a range of spectroscopic methods including mid infrared (MIR) and near-infrared (NIR) spectroscopy [6,7,8,9,10,11,12].

There is an increasing interest among the research and industry communities in the use of molecular spectroscopy methods (e.g., NIR, MIR, Raman) that has been expanded over the past two decades due to the many advantages these techniques offer [6,7,8,9,10,11,12]. Some of these advantages are related to the non-destructive nature of the method, the minimal or no sample preparation required and the speed of the analysis. Techniques using molecular spectroscopy are based upon the overtones and vibrations of the atoms of a molecule when passing infrared (IR) radiation through a tested sample [6,7,8,9,10,11,12]. In the IR region, various fundamental molecular vibrations, including those generated from C-H, O-H, N-H, C=O, and other functional groups can be detected [6,7,8,9,10,11,12]. When a sample is irradiated with IR light, it absorbs the light with frequencies matching characteristic vibrations of particular functional groups, whereas the light of other frequencies will be transmitted or reflected [6,7,8,9,10,11,12]. In this manner, the biochemical components of grapes and wines will determine the amount and frequency of absorbed light and the quantity of reflected or transmitted light can be used to infer the chemical composition of that sample [5,6,7,8,9,10]. Chemical bonds present in the organic matrix of most of the samples vibrate at specific frequencies, which are determined by the mass of the constituent atoms, the shape of the molecule, the stiffness of the bonds, and the periods of the associated vibrational coupling [6,7,8,9,10,11]. A specific vibrational bond is absorbed in the IR spectral region where diatomic molecules have only one bond that may stretch (e.g., the distance between two atoms may increase or decrease).

The use of NIR spectroscopy as an analytical technique is characterised by low molar absorptivity and scattering, which produce a nearly effortless evaluation of a sample [6,7,8,9,10,11]. Spectral “signatures” in the MIR result from the fundamental stretching, bending, and rotating vibrations of the sample molecules, whilst NIR spectra result from complex overtones and high frequency combinations at shorter wavelengths. Spectral peaks in the MIR frequencies are often sharper and better resolved than in the NIR domain, while the higher overtones of the O-H (oxygen-hydrogen), N-H (nitrogen-hydrogen), C-H (carbon-hydrogen) and S-H (sulphur-hydrogen) bands from the MIR wavelengths are still observed in the NIR region, although much weaker than the fundamental frequencies in the MIR. The existence of combination bands (e.g., CO stretch and NH band in protein), gives rise to a crowded NIR spectrum with strongly overlapping bands [6,7,8,9,10,11]. A major disadvantage of this characteristic overlap and complexity in the NIR spectra has been the difficulty of quantification and interpretation of data from NIR spectra. These same characteristics have the advantage that can reduce the need for using a large number of wavelengths during the analysis. In recent years, new instrumentation and computer algorithms have taken advantage of this complexity and have made the technique much more powerful and simple to use [5,6,7,8,9,10]. The MIR region of the electromagnetic spectrum lies between 4000 and 400 cm^−1^ and can be segmented into four broad regions: the X-H stretching region (4000–2500 cm^−1^), the triple bond region (2500–2000 cm^−1^), the double bond region (2000–1500 cm^−1^), and the fingerprint region (1500–400 cm^−1^) [6,7,8,9,10,11]. Such characteristic absorption bands are associated with major components of the sample matrix. Absorptions in the fingerprint region are mainly caused by bending and skeletal vibrations, which are particularly sensitive to large wavenumber shifts, thereby minimising against unambiguous identification of specific functional groups [6,7,8,9,10,11]. The application of FT-MIR in the routine analysis of grapes and wines is of special analytical interest due to the presence of sharp and specific absorption bands for constituents [6,7,8,9,10,11]. With the recent development of sampling accessories attached to a wide range of IR spectrophotometers, such as attenuated total reflectance (ATR) cells, improvements in routine IR analysis, by simplifying sample handling and avoiding measurement problems often found using transmission cells, have been achieved [6,7,8,9,10,11]. In conventional FT-MIR analysis, samples are analysed through a short-path length transmission cell [6,7,8,9,10,11]. The advantage of the transmission cell is that it provides very accurate and reproducible spectroscopic measurements while the main drawbacks are related with issues such as filling and cleaning the cell, variation of sample path length due to window wear and turbidity of the sample. The use of ATR cells minimise or avoid these issues allowing the analysis of a broad range of samples such as grapes and wine juice [6,7,8,9,10,11].

The use of NIR spectroscopy in the wine industry dates back to some early work by Kaffka and Norris while much of the NIR work in the wine industry has concentrated on ethanol analysis [6,7,8,9,10,11,12]. Information about constituents of grape juice and must, as well as wine can be used for management and decision support systems in order to improve, monitor and adapt grape and wine production systems to new challenges. Objective quality measures will allow vineyard managers to target required quality levels and will allow rewarding managers for quality, in terms of quality related grape payment systems where large areas of new plantings coming on stream will apply a correction to the fruit supply and demand situation, placing further urgency on the requirement to determine quality levels. The procedure reported here seems to have much potential for fast and reasonable cost analysis. The results of this work show that the models developed using NIR technology together with chemometric tools allow the quantification of total phenolic compounds and the main families of phenolic compounds in grape skins throughout maturation. 

The objective of this mini review is to present examples and to discuss different applications of visible (VIS), near infrared (NIR) and mid infrared (MIR) to assess and measure phenolic compounds in grape and wines. 

## 2. Analysis of Phenolic Compounds in Grapes, Skins and Seeds

The use of both visible (VIS) and NIR spectroscopy in the wavelength range between 450 and 980 nm was explored to predict anthocyanin in Nebbiolo grapes grown in Italy [13]. Partial least squares (PLS) regression models were developed for ripening parameters and for phenolic ripening indexes in both fresh berries and homogenised samples. Using homogenised grape samples, the authors reported good correlations for ripening index based on phenolic content (R = 0.80) [13]. The use of NIR spectroscopy was also reported to measure total anthocyanins in Cabernet Sauvignon, Carmenere, Merlot, Pinot Noir, and Chardonnay wine grape varieties [14]. These authors also suggested that for the predictions of total anthocyanins, a better reference method should be used to develop robust PLS calibration models for grapes [14]. An optical portable VIS and NIR system (JAZ, Ocean Optics, Dunedin, FL, USA) was reported to predict different ripening parameters in fresh berries [15]. PLS regression was used as method to develop calibration models for extractable anthocyanins yielding a coefficient of determination (R^2^) of 0.74, while less accurate models were obtained for total anthocyanins [15]. Similar results were reported by the same authors using grape samples sourced from different wine regions in Chile [16]. Red grape homogenates (n = 620) were analyzed using a combination of VIS and NIR (400–2500 nm) spectroscopy [17]. The spectra and the analytical data were used to develop PLS calibration models to predict dry matter (DM) content and condensed tannins (CT) [17]. The R^2^ in cross-validation and the SECV were 0.92% and 0.83% w/w for DM and 0.86 and 0.46 mg/g epicatechin equivalents for CT, respectively [17]. The standard error in prediction (SEP) reported for CT was 0.89 mg/g epicatechin equivalent [17].

The potential of NIR spectroscopy to determine the content of phenolic compounds in intact red grapes has been reported and evaluated using a fibre-optic probe as well as a transport quartz cup [18]. Reference values for phenolic compounds were obtained using HPLC-DAD-MS, and modified (M) PLS regression was used as algorithm to develop the quantitative models for flavanols, phenolic acids, anthocyanins and total phenolic compounds [18]. According to these authors, NIR spectroscopy appeared to have an excellent potential for the quantification of total phenolic compounds in grape skins throughout maturation [18]. The validation of these models showed that the best results were obtained for the determination of flavonols (differences between HPLC and NIR of 7.8% using grapes and 10.7% using grape skins) [18]. Good statistics in the external validation were also obtained for the determination of total phenolic compounds (differences of 11.7% using grapes and 14.7% using grape skins) [18].

The feasibility of using FT-NIR spectroscopy to predict the extractable content of phenolic compounds directly in intact grape seeds was reported [19]. Calibration models were based on the correlation of spectral data with the phenolic composition determined by reference chemical methods on 40 grape samples [19]. The effect of season (vintage) was also evaluated and the results showed that the predictive accuracy improved only for spectrophotometric indices when samples from two years were simultaneously considered [19]. The calibration statistics showed that the models developed were sufficiently robust for quantitative purposes in terms of the SEP obtained for total flavonoids, pro-anthocyanidins, low molecular weight flavanols, catechin, epicatechin, procyanidin (SEP < 15%), as well as galloylation percentage [19]. The use of FT-NIR has been also evaluated and compared with instrumental texture parameters associated with the content of total phenols and extractability predictors in intact grape seeds [20]. This study was carried out using Cabernet-Sauvignon seeds from grapes harvested at six different advanced physiological stages throughout ripening and calibrated by flotation to reduce the in-field heterogeneity inside each sample [20]. The best prediction of phenol content in the seeds, performed directly on intact seeds, was found using FT-NIR spectroscopy in transmittance mode [20]. The SEP for total phenol content was less than 8%, while that for phenol extractability was worse [20]. High correlations were reported for the measurement of anthocyanins prediction in Canaiolo grape samples between NIR spectroscopy and total anthocyanis content using PLS regression. These authors reported an excellent performance in cross validation R^2^ of 0.90 and a SECV of 45.15 mg/kg [21]. 

The feasibility of FT-MIR spectroscopy combined with PLS regression to quantify phenolic compounds in red grapes during ripening was reported [22]. The routine reference methods used to quantify these compounds such as total phenolic compounds, total anthocyanins, and condensed tannins were based on UV-VIS spectroscopy [22]. In order to take into account the high natural variability of grapes when building the calibration models, the authors collected fresh grapes from six varieties, at different phenolic ripening states, which were harvested during three vintages [22]. The statistics reported by the authors for the prediction of total phenolic were a root mean square error of prediction (RMSEP) of 4.3% and a ratio deviation in prediction (RPD) of 4.5, for total anthocyanins RMSEP of 5.9% and RPD of 3.5, and for condensed tannins RMSEP of 5.8% and RPD of 3.8 [22]. In another study, procyanidins were extracted with a mixture of methanol and acetone in water from seeds sourced from white and red wine grape varieties and analysed using FT-MIR spectroscopy [23]. A fractionation by graded methanol/chloroform precipitations produced a total of 26 samples that were characterised using thiolysis as pre-treatment followed by HPLC-UV and MS detection [23]. The average degree of polymerisation (DPn) of the procyanidins in the samples ranged from 2–11 flavan-3-ol residues [23]. PLS regression models for the determination of DPn, yield a RMSECV of 11.7%, with a R^2^ of 0.91 and a RMSEP of 2.58 [23]. According to the authors, the application of orthogonal projection to latent structures (O-PLS) improves the interpretation of the regression models [23]. 

In recent years, new applications of VIS and NIR have been developed by means of hyperspectral imaging [24,25,26,27,28]. Hyperspectral images of intact grapes during ripening were recorded using a NIR hyperspectral imaging system (900–1700 nm) [24]. Spectral data were correlated with grape skin total phenolic concentration using MPLS regression, as well as using different spectral pre-treatments [24]. The calibration statistics obtained using MPLS and a combination of red and white wine grape samples were R^2^ and SECV of 0.89 and 1.23 mg/g for total phenolic concentration [24]. Separate calibration models for red and white grape samples were also developed and compared by the authors [24]. The results obtained showed a good potential for a fast and inexpensive screening of these parameters in intact grapes [24]. The determination of the total anthocyanin content in skins of Cabernet Sauvignon grapes produced in Shaanxi province (China) using hyperspectral imaging was reported [25]. The hyperspectral images of intact grapes during ripening were collected using NIR hyperspectral imaging covering the spectral range between 900 and 1700 nm [25]. Calibrations were developed using MPLS as an algorithm, and an R of 0.86 and SEP values of 2.62 and 3.05 mg/g for the measurement of non-acylated and total anthocyanins in wine grape skin samples were reported by the authors [25]. NIR hyperspectral imaging has been used to determine flavanols in seeds of red (cv. Tempranillo) and white (cv. Zalema) grapes [26,27]. As reference measurements, the flavanol content was estimated using the p-dimethylaminocinnamaldehyde (DMACA) method [26,27]. Not only total flavanol content was evaluated but also the quantity of flavanols that would be extracted into the wine during winemaking [26,27]. Calibrations were developed using PLS regression yielding a R^2^ of 0.73 for total flavanol content and R^2^ of 0.85 for flavanols extracted using a model wine solution. Higher R^2^ values (0.88) were reported by the authors when grape cultivars were analysed separately [26,27]. The potential of NIR hyperspectral imaging to determine anthocyanins in intact grape has been evaluated [28]. The R^2^ values reported for the concentration of total anthocyanins in red grapes were 0.65. According to the authors, the correlation value obtained was better than the value reported in another recent scientific work which estimated anthocyanin values grapes form the Cabernet Sauvignon variety [28]. 

## 3. Analysis of Phenolic Compounds in Wine

The combination of FT-MIR and attenuated total reflectance (ATR) was explored for the determination of total phenolic, flavonoid content and antioxidant capacity (DPPH and FRAP assays) in Moscatel dessert wines (n = 56) [29]. Prediction models were developed for the referred parameters using PLS regression. The R^2^ values in the calibration models ranged from 0.67–0.87 [29]. The root mean square errors of calibration (RMSEC) and cross validation (RMSECV) as well as the relative errors of prediction (REP) were calculated [29]. The minimum errors of prediction were obtained for total flavonoid content (0.2%) and maximum values (22%) for antioxidant capacity. The authors concluded that the proposed method may be used for rapid screening of total phenolic and flavonoid contents in Moscatel dessert wines [29].

The use of FT-MIR spectroscopy combined with chemometrics was also evaluated as method for correlating the spectral response of a sample to its compositional phenolic profile, as a valid alternative method to the standard UV-VIS technique [30]. In this study, the evaluation of FT-MIR combined with PLS regression was reported for the determination of 12 anthocyanins (3-O-glucosides of delphinidin, cyanidin, petunidin, peonidin and malvidin, as well as acetic acid esters and p-coumaric acid esters of petunidin, peonidin and malvidin and caffeic acid ester of malvidin) and three sums (sum of non-acylated anthocyanins, sum of acetylated anthocyanins and sum of coumaroylated anthocyanins) in red wines [31]. Reference values of anthocyanin concentrations by reverse-phase HPLC-DAD were used to calibrate the models [31]. A principal component analysis (PCA) method was applied to the reference values where a differentiation of wine samples by wine type (young wines of 2005, young wines of 2004 and crianza and reserva wines) was reported by the authors [31]. Most of the anthocyanins and their sums have been predicted with a SEP of 15%–30% for young wines. The results reported by the authors suggested that the model built using FT-MIR spectroscopy was adequate for the rapid determination of total anthocyanin content in young wines of the current vintage. However, a careful robust external validation of the technique is required in order to maintain the prediction errors within control limits for routine analysis [31]. Color components of commercial red wines such as total wine color, polymeric pigments, total anthocyanins, and copigmentation index were investigated using FT-MIR spectroscopy [32]. The composition of red wines showed great difference in terms of total color (5.07 +/− 1.95 AU at 520 nm) compared to the copigmentation index (0.66 +/− 0.58 AU at 520 nm). The prediction of total wine color, total anthocyanins, and polymeric pigments showed a good correlation of R^2^ 0.82; however, the copigmentation index yielded low correlation coefficients (R^2^ 0.57) [32].

A rapid method to quantify phenolic compounds all during the red winemaking process using FT-MIR spectroscopy and chemometrics was reported [33]. Reference values were obtained using UV-VIS spectroscopy, where total phenolic compounds (TPC), total anthocyanins (TA), and condensed tannins (CT) were measured [33]. The spectral regions selected by the authors for each model were between 979 and 2989 cm^−1^, and the optimized calibration models yield good calibration statistics for the different parameters evaluated (R^2^ > 0.95 and RPD > 4.0 for TPC; R^2^ > 0.90 and RPD > 3.0 for TA; R^2^ < 0.8 and RPD < 3.0 for CT). It was concluded by the authors that FT-MIR spectroscopy together with multivariate calibration could be a rapid and valuable tool for wineries to carry out the monitoring of phenolic compound extraction during winemaking [33]. 

The ability of an electronic tongue (ET) based on FT-MIR spectroscopy as a gustative sensor was assessed by emulating the responses of a tasting panel for the gustative mouthfeel “tannin amount” [34]. The FT-MIR spectra were modeled against the sensory responses evaluated in 37 red wines by means of PLS regression models. According to the authors, the iterative predictor weighting IPW-PLS technique showed the best results with the smallest RMSEC and RMSECV values (0.07 and 0.13, respectively) using 20 selected wavenumbers [34]. The use of spectroscopic analysis shows that Madeira wine age, produced from a known grape variety, can be predicted with good accuracy from its volatile and phenolic composition, as well as from the spectra collected in a UV-VIS instrument [35]. The PLS regression models estimated were able to predict wine age with a RMSECV of 0.9, 1.1, and 1.4 years, respectively. The sample-specific prediction intervals computed also allowed for the analysis of differences between observed and predicted values, and confirmed the interesting wine age prediction abilities of the proposed methodologies [35]. According to the authors, a compromise between model accuracy and cost of analysis can be reached in order to decide which methodology to use [35]. As a function of a particular application scenario, the more time-consuming and complex techniques such as GC-MS or HPLC-DAD delivered more accurate results [35]. However, satisfactory calibration and prediction statistics were obtained using UV-VIS spectroscopy as an analytical method [35]. The use of FT-MIR spectroscopy allows fast measurement of different wine components, but quantification of tannins is difficult due to interferences from spectral responses of other wine components [36]. Four different variable selection tools were investigated for the identification of the most important spectral regions which would allow quantification of tannins from the spectra using PLS regression. The study included the development of a new variable selection tool, iterative backward elimination of changeable size intervals PLS regression. The spectral regions identified by the different variable selection methods were not identical, but all included two regions (1485–1425 and 1060–995 cm^−1^). The spectral regions identified from the variable selection methods were used to develop calibration models. All four variable selection methods identified regions that allowed an improved quantitative prediction of tannins (RMSEP = 69–79 mg of CE/L; R= 0.93–0.94) as compared to a calibration model developed using all variables (RMSEP = 115 mg of CE/L; R = 0.87) [36].

Spectral analysis based on UV-VIS-NIR was reported as a method to analyse wine [37]. In this study, the authors determined trans-resveratrol, oenin, malvin, catechin, epicatechin, quercetin and syringic acid in commercial red wines from two Spanish regions, namely DO Rias Baixas and DO Ribeira Sacra [37]. Calibration models were developed using principal component regression (PCR) or PLS regression and HPLC as a reference method [37]. The results from this study showed that good calibration statistics using PLS regression models were obtained to quantify all polyphenol compounds from the Rias Baixas wines [37]. Intermediate PLS calibration statistics were obtained for the measurement of quercetin, epicatechin, oenin and syringic acid in wines sourced from Ribeira Sacra, and for catechin and oenin in red wines obtained using Mencia grapes [37].

**Figure 1:**
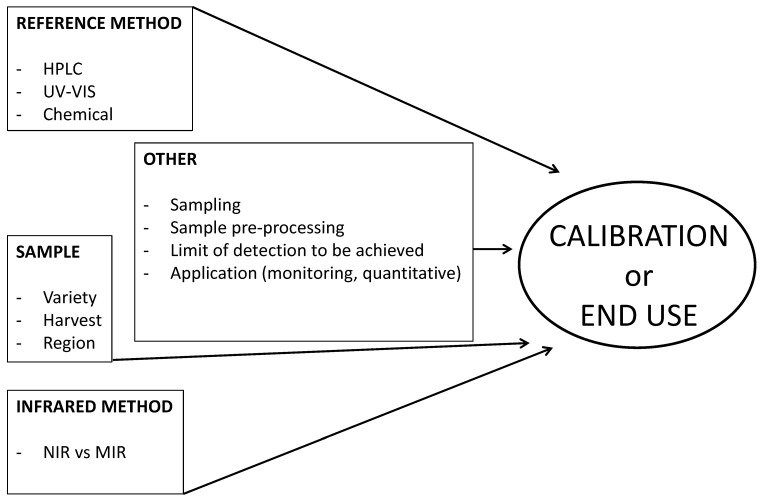
Factors that affect the end use of infrared methods for the analysis of phenolic compounds in grapes and wine samples.

The composition of phenolic compounds plays an important role in food science and nutrition; thus, there is need for a new method of analysis that is able to speed up the monitoring of product quality parameters [38]. The use of FT-MIR spectroscopy was also evaluated to measure the total antioxidant activity (TAC) of red wines. The PLS regression models showed a good predictive ability (R = 0.85) of the antioxidant activity of red wines in cross validation [38]. These authors concluded that FT-MIR spectroscopy is a promising technique to rapidly provide information on TAC of red wines and has a high potential to be implemented for the rapid screening of TAC during winemaking [38]. Tannin content and composition are critical quality components of red wines [39]. However, few spectroscopic methods assessing these phenols in wine have been described [39]. The use of FT-MIR combined with chemometric techniques was evaluated for the quantitative analysis of red wine tannins [39]. Calibration models were developed using protein precipitation and phloroglucinolysis as analytical reference methods, and after spectra preprocessing, six different predictive PLS models were evaluated by the authors [39]. The best calibration models were obtained for tannin concentration (RMSEC = 2.6%, RMSEP = 9.4%, R = 0.995) and for the prediction of the mean degree of polymerization (mDP) of the tannins (RMSEC = 6.7%, RMSEP = 10.3%, R = 0.958) [39]. Figure 1 summarises the main factors or variables affecting the ability of IR spectroscopy to analyse phenolic compounds in grape and wine samples.

## 4. Concluding Remarks

The major advances in the application of spectroscopic techniques for the analysis of phenolic compounds in grapes and wines has been related to the development of powerful mathematical techniques known collectively as chemometrics. These data analysis methods allow the extraction of valuable information from large and complex data sets, underpinning the application of methods based on spectroscopy (e.g., NIR, MIR, UV, VIS). As fast and easy-to-operate techniques, spectroscopy has already gained wide industrial acceptance for routine analysis in the grape and wine industries. However, several critical aspects and limitations still exist, associated with instrument availability, type of application (e.g., grape, juice, wine) and the overall understanding of the technology. For example, one of the main factors that determine the type of instrument to be selected by the industry is the type of sample and parameter to be measured. This simple issue plays an important role in the success of a given application in determining the accuracy of the results obtained. 

Although, spectroscopy generally cannot measure molecules with low concentration, the indirect effects of such differences in the whole matrix of grape and wine samples can be observed or assessed in the spectrum of a given sample (e.g., fingerprint). This fingerprint, with the application of chemometric techniques (e.g., principal component analysis or discriminant analysis, PLS), can be used to elucidate particular compositional characteristics associated with phenolic compounds in grapes and wine samples not easily detected by traditional targeted chemical analysis. 
